# Borderline Arrhythmogenic Cardiomyopathy in an Athlete: Exercise Testing May Support Clinical Decision-Making

**DOI:** 10.1016/j.jaccas.2026.107837

**Published:** 2026-04-16

**Authors:** Simon Wernhart, Martin Halle, Mark J. Haykowsky, Satyam Sarma

**Affiliations:** a Department for Preventive Sports Medicine and Sports Cardiology, TUM School of Medicine and Health, TUM University Hospital, Technical University of Munich (TUM), Munich, Germany; b DZHK (German Centre for Cardiovascular Research), Partner Site Munich Heart Alliance, Munich, Germany; c Integrated Cardiovascular and Exercise Physiology and Rehabilitation (iCARE) Laboratory, College of Health Sciences, University of Alberta, Edmonton, Alberta, Canada; d Hochgebirgsklinik, Davos, Switzerland; e Department of Internal Medicine, University of Texas Southwestern Medical Center, Dallas, Texas, USA; f Institute for Exercise and Environmental Medicine, Texas Health Presbyterian Hospital Dallas, Dallas, Texas, USA

**Keywords:** arrhythmogenic cardiomyopathy, athlete, exercise stress testing

## Abstract

**Background:**

Arrhythmogenic cardiomyopathy (ACM) bears a considerable risk of malignant arrhythmias and heart failure, but diagnosis in athletes can be challenging.

**Case Summary:**

We present a male 52-year-old long-distance runner with palpitations, a dilated right ventricle with apical akinesia, nonsustained ventricular tachycardia, and negative genetic testing. We performed combined stress echocardiography and cardiopulmonary exercise testing showing blunted exercise response and a decrease in right ventricular strain during exercise. Although the modified task force criteria were not met, a diagnosis of ACM based on impaired exercise response was made, and termination of high-intensity endurance and restriction to 150 minutes/wk of moderate-intensity training was recommended.

**Discussion:**

The modified task force criteria are recommended for clinical decision-making, but clinical uncertainties remain in gene-elusive cases. Stress testing may complement current guidelines to unmask underlying cardiomyopathy.

**Take-Home Message:**

Stress testing should be considered in athletes showing borderline features of ACM.

## Visual Summary

**Figure:**
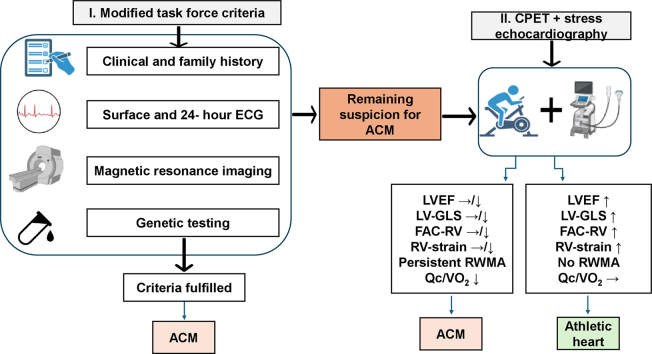

## History of Presentation

A 52-year-old male long-distance runner consulted us for sports cardiological advice in 2025 after experiencing palpitations in 2013. He reported a steady decline in exercise tolerance starting in 2011 but denied shortness of breath, syncope, or chest pain. Physical examination revealed no abnormalities.

**Take-Home Messages**

• This case highlights the potential benefit of exercise stress testing to reveal blunted cardiac response during exercise.
• Combined stress echocardiography and cardiopulmonary exercise testing may contribute to clinical decision-making in borderline arrhythmogenic cardiomyopathy.

## Past Medical History

There was no previous medical or family history of sudden cardiac death. Given recurrent palpitations in the presence of a normal electrocardiogram (ECG) and a dilated right ventricle (RV) on echocardiography in 2013, the patient was referred for magnetic resonance imaging (MRI) by the treating cardiologist, which did not demonstrate late gadolinium enhancement (LGE) (Video 1A). There was RV dilatation (end-diastolic volume: 157 mL/m^2^), but not of the left ventricle (LV; end-diastolic volume: 107 mL/m^2^). LV and RV ejection fractions were slightly reduced (53% and 39%, respectively), and the RV demonstrated microaneurysms along the midventricular RV free wall and regional wall motion abnormality of the RV apex. An anaphylactoid reaction to gadolinium necessitated premature termination of the examination and prevented follow-up MRI. Unfortunately, fat-suppression techniques were not available for detecting fibrofatty infiltration. A loop recorder was implanted in 2014. The beta-blocker naïve patient showed 1 episode of a 7-beat nonsustained ventricular tachycardia (VT) during the follow-up period from 2014 to 2017. Unfortunately, the patient wanted the loop recorder removed because of muscular strain. A physician himself, the patient reduced his training volume to 5 h/wk of low-intensity endurance training (intensity of 11-12 on the Borg scale) and did not have cardiological follow-up until his consultation in our department in 2025.

**Visual Summary:**
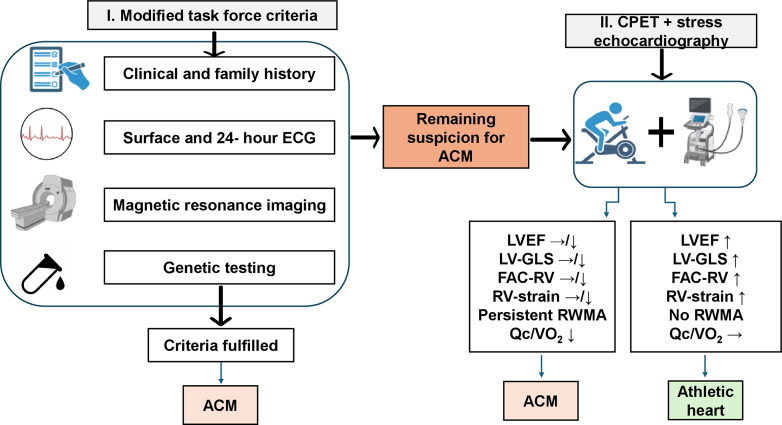
Proposed Diagnostic Algorithm for Athletes With Borderline Arrhythmogenic Cardiomyopathy The initial algorithm refers to the modified task force criteria,^1^ including clinical and family history, surface and 24-hour Holter electrocardiogram (ECG), and magnetic resonance imaging and genetic testing. If diagnosis of arrhythmogenic cardiomyopathy (ACM) can be made, no further testing is required. If diagnostic criteria are borderline, we recommend additional stress echocardiography combined with cardiopulmonary exercise testing (CPET). ACM may be considered in case of a lack of increase or decline in left ventricular ejection fraction (LVEF), left ventricular global longitudinal strain (LV-GLS), fractional area change of the right ventricle (FAC-RV), right ventricular global strain (RV-strain), or regional wall motion abnormalities (RWMA). A reduced cardiac output (Q_c_) to oxygen consumption (Vo_2_) slope may suggest reduced cardiac reserve compatible with ACM rather than exercise-induced adaptations. In the presence of an increase in contractility (LVEF, LV-GLS, RV-FAC, RV-strain) and absence of RWMA, physiological adaptations should be suspected. Images were created with biorender.com.

## Differential Diagnosis

This case raises suspicion for ACM, showing morphological changes on MRI with wall motion abnormalities of the dilated RV (Video 1A) and a history of VT. Despite the absence of LGE and the lack of traditional cardiovascular risk factors, previous myocarditis and coronary artery disease need to be considered in the presence of regional wall motion abnormalities. Physiological exercise-induced adaptations of the heart also need to be considered as a reason for RV dilatation.

## Investigations

**Figure 1:**
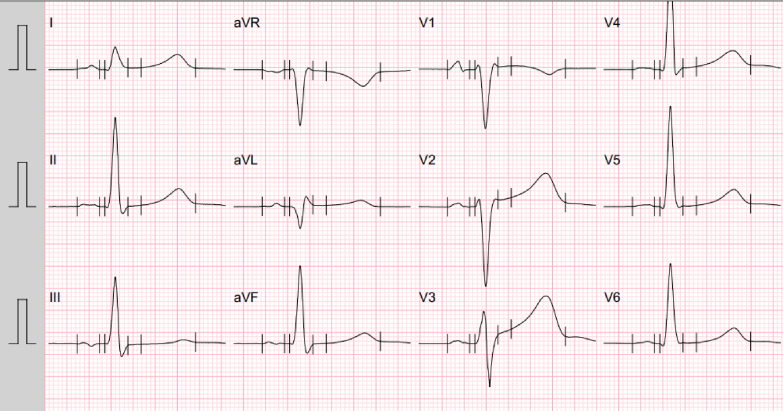
Signal-Averaged Resting 12-Lead Electrocardiogram of the Patient

After 10 uneventful years, the ECG showed regular sinus rhythm without T-wave inversions (Figure 1) in 2025. Holter monitoring revealed less than 50 premature ventricular beats over 24 hours, and echocardiography showed preserved LV ejection fraction (56%) and slightly reduced RV ejection fraction (RV fractional area change: 41%), with apical akinesia in an atypical RV view and low-normal RV strain (free wall: −22.3%; global RV: −20.4%) (Videos 1B and 1C). We initiated genetic testing, which remained elusive (Table 1).

**Table 1:** Gene Panel for Genetic Testing in the Patient

Plakophilin (PKP2)
Desmoplakin (DSP)
Desmocollin 2 (DSC2)
Desmoglein 2 (DSG2)
Plakoglobin (JUP)
Transmembrane protein 43 (TMEM 43)
Phospholamban (PLN)
Filamin C (FLNC)
Desmin (DES)
Lamin A/C (LMNA)
Transforming growth factor-3 (TGFB3)
Alpha-T-catenin (CTNNA3)
Cadherin-2 (CDH2)
Sodium channel alpha unit (SCN5A)

**Figure 2:**
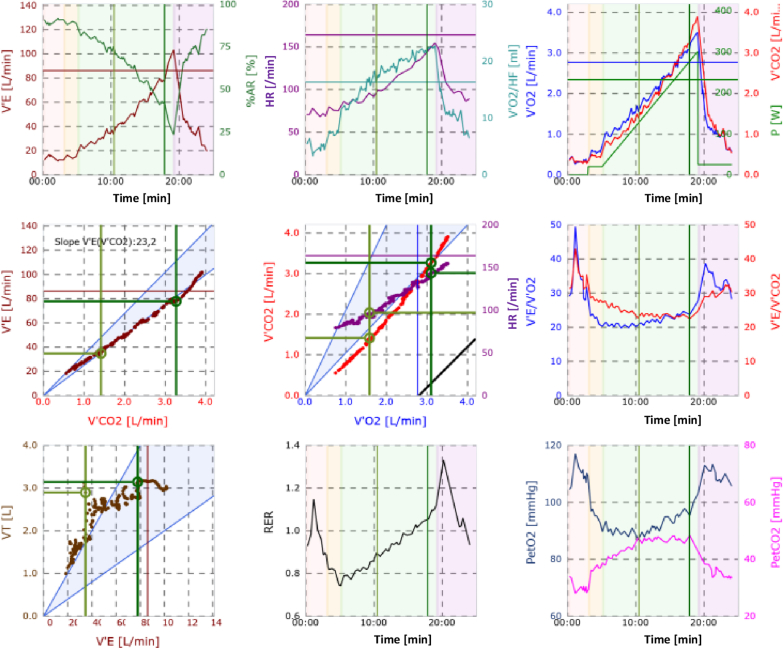
9-Panel Plot of Cardiopulmonary Exercise Testing HR = heart rate; RER = respiratory exchange ratio; PETco_2_ = end-tidal carbon dioxide; PETo_2_ = end-tidal oxygen; Vco_2_ = carbon dioxide production; VE = minute ventilation; Vo_2_ = oxygen uptake.

Based on current diagnostic criteria,^1^ the patient displayed 1 definite major criterion (RV dilatation and regional wall motion abnormality on MRI) and 1 criterion for reported VT, but without a rhythm strip to determine RV morphology. Given the inconclusive results and inability to perform another MRI, we performed semisupine exercise stress echocardiography combined with cardiopulmonary exercise testing (CPET) (Figure 2), up to 275 W and observed 6 polymorphic premature ventricular beats from the RV. Heart rate and blood pressure response to exercise were within normal range. Stress echocardiography demonstrated a slight increase in RV fractional area change (45%) but a decline in RV strain (change from rest to peak: lateral free wall, −6.3%; global RV strain, −10.5%) and wall motion abnormalities of the RV apex, while LV ejection fraction increased to 66% (Videos 1D and 1E). Cardiac output (Q_c_), measured with echocardiography, increased from 5.0 L/min at rest (stroke volume: 85 mL, heart rate: 59 beats/min, LV end-diastolic volume: 162 mL) to 19.0 L/min during peak exercise (stroke volume:113 mL, heart rate: 168 beats/min, LV end-diastolic volume: 171 mL). CPET revealed an oxygen consumption (Vo_2_) at peak exercise of 41.6 mL/min/kg (135% of predicted). The Q_c_/Vo_2_ slope was slightly reduced compared to the literature (4.48; normal: 5-6), potentially indicating blunted cardiac response to exercise.^2^

**EQUIPMENT LIST:** Equipment List of Combined Cardiopulmonary Exercise Testing and Stress Echocardiography

Imaging • Transthoracic echocardiography (Philips Healthcare) ○ X5-1c transducer • Stress echocardiography ○ Ergoselect 1200 (Ergoline Moving to Health) ○ Tilt the patient backwards and sidewards to gain adequate image quality for the main planes of standard echocardiography Cardiopulmonary exercise testing • Electrodes for the electrocardiogram ○ Ambu Blue Sensor VL-00-S electrodes • Metabolic chart ○ Cosmed (The Metabolic Company) • Masks ○ Hans Rudolph ○ Size adapted to patient size • Exercise protocol ○ Estimate the expected performance of the patient and aim for an exercise duration of 8 to 12 min ○ Start with 20 W and use an incline of 10 or 20 W/min depending on expected patient performance ○ Aim for metabolic exertion with a ratio of carbon dioxide production to oxygen consumption of at least 1.05

## Management

Given borderline ACM criteria, with evidence for exercise-induced reduction of RV strain, persistent wall motion abnormalities, and slightly reduced Q_c_/Vo_2_, we opted for a clinical diagnosis of ACM and prescribed low-dose beta-blocker therapy. Exercise intensity was limited to heart rates below the first ventilatory threshold. Duration of weekly endurance training was limited to 150 minutes.^3^ We also recommended low-intensity dynamic resistance training twice weekly. In the absence of syncopes, sustained VT, or pathogenic gene variants, we opted against primary prophylactic placement of an implantable cardioverter-defibrillator (ICD). The patient did not wish to receive another loop recorder, and we decided to use smartwatch monitoring with the option to trigger ECGs whenever symptoms occur.

## Outcome and Follow-Up

In the absence of symptoms, we performed annual follow-up including echocardiography, exercise stress testing, Holter monitoring, and assessment of smartwatch documentation, which have been insignificant.

## Discussion

ACM has been associated with sudden cardiac death during sports,^4^ and the clinical diagnosis is based on modified task force criteria.^1^ Endurance athletes may present with RV dilatation compatible with minor or even major criteria for ACM, outlining the overlap between exercise-induced adaptations and cardiomyopathic heart and necessitating repetitive examination.^5^ Our patient exceeded suggested cutoff volumes for exercise-induced RV adaptations,^6^ which may favor ACM in the presence of regional wall motion abnormalities.

Early diagnosis of ACM is pivotal, as disease progression has been associated with high-intensity endurance sports.^3^^,^^6^ Current ACM criteria do not incorporate exercise testing, which may provide valuable information on exercise-induced ventricular reserve in patients with equivocal findings. Our finding of a change in RV free wall strain to less negative values during exercise (though not diagnostic) is supported by a recent study showing a cutoff value for RV free wall strain of greater than −2.35% at moderate exercise intensity compared to rest in order to identify exercise-induced ACM.^7^ Exercise-induced attenuation of RV measures, such as RV end-systolic pressure-area ratio or RV fractional area change, hold promise to identify athletes with RV arrhythmias^8^ and support the concept of stress testing in borderline cases.

It is intriguing that there was no segmental difference in RV strain on resting echocardiography. This may be explained by the circumscribed regional wall motion abnormalities, which may not have been caught in the regular 4-chamber view. Atypical views (Video 1D) may be necessary to capture the complex RV architecture. However, augmentation of LV ejection fraction from 56% to 66% is consistent with preserved reserve^9^ and does not suggest LV involvement. ACM rather than myocarditis may be the most likely diagnosis in the absence of prior clinically apparent infectious syndromes and RV-LGE on MRI. However, absence of RV-LGE does not necessarily exclude the presence of scar, particularly given the thin-walled nature of the RV and the limited spatial resolution of current MRI techniques.

In the present case, exercise imaging was of clinical value to counsel the patient against continuing high-intensity endurance sports. The patient had exercised between 25 and 30 MET h/wk for 2 decades, which may have facilitated the development of exercise-induced ACM.^5^^,^^10^, ^11^, ^12^

The vector of VT is important, which may either present a minor or major criterion. Patients with suspicion of ACM but no indication for ICD implantation may receive loop recorders or smartwatch monitoring, which do not provide information on the vector of arrhythmias. As loop recorders are not reimbursed in our country, smartwatches may be a cost-effective and feasible approach for patient-triggered ECG whenever symptoms occur. We implement ECG data of borderline ACM into our telemetric unit, enabling cardiologists to monitor such patients more closely. Although 1 episode of nonsustained VT in our patient did not lead to ICD recommendation, sustained VT may change our approach. This is supported by data showing that athletes may experience nonsustained VT in the absence of cardiomyopathy.^13^

Longitudinal studies have illustrated that persistent ventricular strain induced by continued high-intensity endurance sports can increase the arrhythmogenic burden and may lead to an earlier onset of the disease in athletes, even in the absence of a positive genotype.^10^^,^^11^^,^^14^ Risk stratification of borderline and gene-elusive individuals could be expanded by integrating dynamic assessment of the RV during exercise. If the diagnosis remains unclear after extended stress testing, electrophysiological evaluation may be useful to tailor exercise recommendation and refine risk stratification. This is supported by findings that inducibility of arrhythmias bears prognostic potential in this population.^11^ Electroanatomical mapping may help to identify the subtle RV substrate not detectable on MRI, fostering earlier diagnosis and potential therapeutic options.^15^

The Q_c_/VO_2_ slope may be a promising parameter to assess whether cardiac output matches metabolic demand and has been reported to be consistent in most patients.^2^ While our patient demonstrated lower values than reported in the general population, this warrants validation in a larger cohort of athletes.

Overall, our case demonstrates the complexity of gene-elusive probable ACM, which should induce meticulous follow-up: 1) annual cardiological follow-ups including echocardiography, stress testing, and rhythm monitoring, and (if feasible) repeat MRI scans to detect LGE; 2) discussion of repetitive genetic testing over time; 3) exercise ECG to analyze premature ventricular beat burden and complexity during and after exercise; and 4) exclusion from competitive, high-intensity sports, which may lead to stigmatization of athletes, necessitating shared decision-making between physician and athlete.

## Conclusions

Dealing with athletes fulfilling borderline criteria for gene-elusive ACM remains challenging and requires a better understanding of exercise physiology during different types of exercise. Combining stress echocardiography and CPET may contribute to this task.

## Funding Support and Author Disclosures

Dr Haykowsky is funded in part by a Research Chair in Aging in the College of Health Sciences, Faculty of Nursing. Dr Wernhart has received honoraria for lectures from Bristol-Myers Squibb. Dr Halle reports honoraria for lectures from Abbott, Amgen, Astra-Zeneca, Boehringer-Ingelheim, BMW, Bristol-Myers Squibb, Daiichi-Sankyo, Lilly, Medi, MSD Sharp & Dohme GmbH, Norsan, Novartis, Pfizer, and Roche; and consulting fees from Medical Park. All other authors have reported that they have no relationships relevant to the contents of this paper to disclose.

## Appendix

Video 1 Resting and Exercise Images of the Athlete (A) Four-chamber view of magnetic resonance imaging at initial presentation in an external clinic. (B) Four-chamber view of the resting echocardiogram showing a dilated right ventricle with slightly reduced ejection fraction. (C) Four-chamber view of resting echocardiography with low normal right ventricular strain (lateral free wall: −22.3%, global right ventricular strain: −20.4%; more negative values depict better contractile function). (D) Right-dominant 4-chamber view during peak exercise (275 W) showing an apical akinetic, aneurysmatic segment. (E) Right ventricular strain during peak exercise (275 W) showing lower right ventricular strain compared to rest (lateral free wall: −6.3%, global right ventricular strain: −10.5%).
